# Editorial: Novel methods and technologies for the evaluation of drug outcomes and policies

**DOI:** 10.3389/fphar.2024.1396034

**Published:** 2024-04-18

**Authors:** Blythe Adamson, Amr Makady, Grammati Sarri, Omneya Mohamed, Zaheer Babar, Dalia M. Dawoud

**Affiliations:** ^1^ Flatiron Health, New York, NY, United States; ^2^ The Comparative Health Outcomes, Policy and Economics (CHOICE) Institute, University of Washington, Seattle, WA, United States; ^3^ Janssen-Cilag B.V., Breda, Netherlands; ^4^ Cytel, London, United Kingdom; ^5^ Baxter AG Scientific Office, Dubai, United Arab Emirates; ^6^ Department of Pharmacy, University of Huddersfield, Huddersfield, United Kingdom; ^7^ Faculty of Pharmacy, Cairo University, Cairo, Egypt; ^8^ National Institute for Health and Care Excellence (NICE), London, United Kingdom

**Keywords:** methods, analytic, artificial intelligence, causal inference, health technology assessment (HTA), HEOR

Globally, providing quality, equitable healthcare by accelerating patient access to new, promising health technologies while balancing the impact of their increased expenditures remains a global challenge. In parallel, the landscape of techniques and tools available to evaluate the safety and effectiveness of drugs is rapidly evolving with the advent of novel technologies and methodologies, thereby re-inventing the way we evaluate health outcomes and policies. This Research Topic of Frontiers in Pharmacology presents a compelling collection of scientific papers that delve into these advancements, offering insights into the latest developments in this dynamic field.

Artificial intelligence (AI) and machine learning (ML) methodologies are key themes explored in three papers within this Research Topic. The study by Zitu et al. on the generalizability of ML methods in detecting adverse drug events from clinical narratives in electronic medical records is a testament to the potential of AI in enhancing drug safety monitoring. Adamson et al. application of AI and ML in extracting real-world data from electronic health records (EHRs) is a stride forward in oncology research. This approach exemplifies how technology can enhance the curation of health records into valuable data sources. Vithlani et al. systematically review the conduct and reporting of health economic evaluations for AI-based healthcare interventions. Their work reveals the rapid growth in this area and the necessity for specific reporting standards to enhance transparency and decision-making in AI intervention evaluations. Some believe the increasing use of AI and ML raises ethical concerns regarding data privacy, bias, and transparency.

There was vital discussion around dominant Research Topic in Health Technology Assessment (HTA); integrating real-world evidence (RWE) in HTA, expanding analytical approaches (cost-effectiveness, equity-informed analyses) to include considerations beyond clinical and economic value drivers, and exploring new, reactive HTA approaches. Claire et al. bring forward insights, drawn from a multi-stakeholder workshop, to address the slow adoption of RWE in HTA compared to regulatory processes and the underlying reasons for staying behind. They emphasize the need for developing resources to promote best practices for conducting RWE studies, comprehensive training, stakeholder collaboration, and impactful research projects to bridge this gap, thereby enhancing HTA’s evidence base for informed healthcare decisions. Muir et al. review on integrating additional value elements in HTA modeling methods is a call to broaden the scope of health technology assessments. By incorporating societal values and health equity, their work advocates for a more holistic approach to evaluating new therapies. In the same direction, Zebrowska et al. and team’s groundbreaking work on quantifying the impact of novel metastatic cancer therapies on health inequalities is a sobering reminder of the disparities in healthcare by offering an example of equity-informed analysis. Their study highlights how advancements in treatments may inadvertently widen the survival gap among different patient groups, emphasizing the need for more equitable healthcare solutions.

Cheyne et al. draw parallels between “living” clinical practice guidelines and HTA. Their reflections on incorporating continuous evidence synthesis in HTA processes offer a new paradigm in healthcare evaluation, ensuring that HTA remains responsive and current in a rapidly evolving evidence landscape.

Moving to Research Topic on advanced RWD analysis techniques in health economics and outcomes research (HEOR), the selected articles presented solutions for evaluating effectiveness and safety for new drugs in rare and very rare diseases and presented case study applications in causal inference and pharmacoepidemiology. Mackay and Springford advocacy for Bayesian methods in evaluating treatments for rare indications addresses a critical gap in HEOR. They argue for the use of Bayesian approaches to overcome challenges in small sample sizes and disconnected evidence networks, paving the way for more nuanced and robust analysis in rare disease settings. Franchini et al. Introduce an innovative approach in discrete event simulation, focusing on event-specific probabilities and distributions, especially in the context of censored data. Their methodological advancements in modeling competing events hold significant promise for more accurate and nuanced analysis in pharmacoeconomic studies.

Causal inference principles applied by Polito et al. and team to external control analysis in observational data is a noteworthy contribution. By defining the estimand attributes and selecting appropriate estimators, their study offers a refined approach to evaluating long-term survival outcomes in metastatic non-small cell lung cancer. Jeong et al. and team’s use of network analysis to elucidate the dynamic landscape of drug-drug interactions offers a novel perspective. Their work underscores the potential of computational methods in identifying key research areas and informing clinical practice. The analysis of the FDA Adverse Event Reporting System by Su et al. offers a deep dive into the adverse event profiles of Denosumab and Zoledronic acid. Their findings provide invaluable insights for clinicians and policymakers, highlighting the importance of ongoing safety monitoring in pharmacovigilance.

Finally, the living systematic review by Elvidge et al. provides a crucial update on the economic evaluations of COVID-19 diagnostics and treatments emphasizing the need for a real time, regularly updated decision-making. Two years into the pandemic, their work synthesizes cost-effectiveness evidence for various interventions, highlighting the importance of making informed healthcare decisions in the rapidly changing landscape of COVID-19 management as new data emerges. This study underscores the ongoing need for living robust economic evaluations in guiding healthcare strategies, especially in a rapidly-changing pandemic setting.

This Research Topic not only reflects the recent trends in HEOR and rapid advancements in drug evaluation and policy research but also underscores the need for continuous adaptation and integration of novel methods in healthcare decision-making. It remains a potential challenge in accessing and implementing novel technologies, particularly in resource-limited settings. From the economic evaluations of emerging therapies to the cutting-edge use of AI and ML in data analysis, these studies collectively push the boundaries of current knowledge, paving the way for more informed, efficient, and equitable healthcare systems.

## Supplementary material

SUPPLEMENTARY FIGURE S1 Art by Catherine Au Yeung.

