# Supplementary figures and images for: Editorial: Novel methods and technologies for the evaluation of drug outcomes and policies
Source: Front Pharmacol. 2024 Apr 18;15:1396034. doi: 10.3389/fphar.2024.1396034 (PMC11063715; doi:10.3389/fphar.2024.1396034)

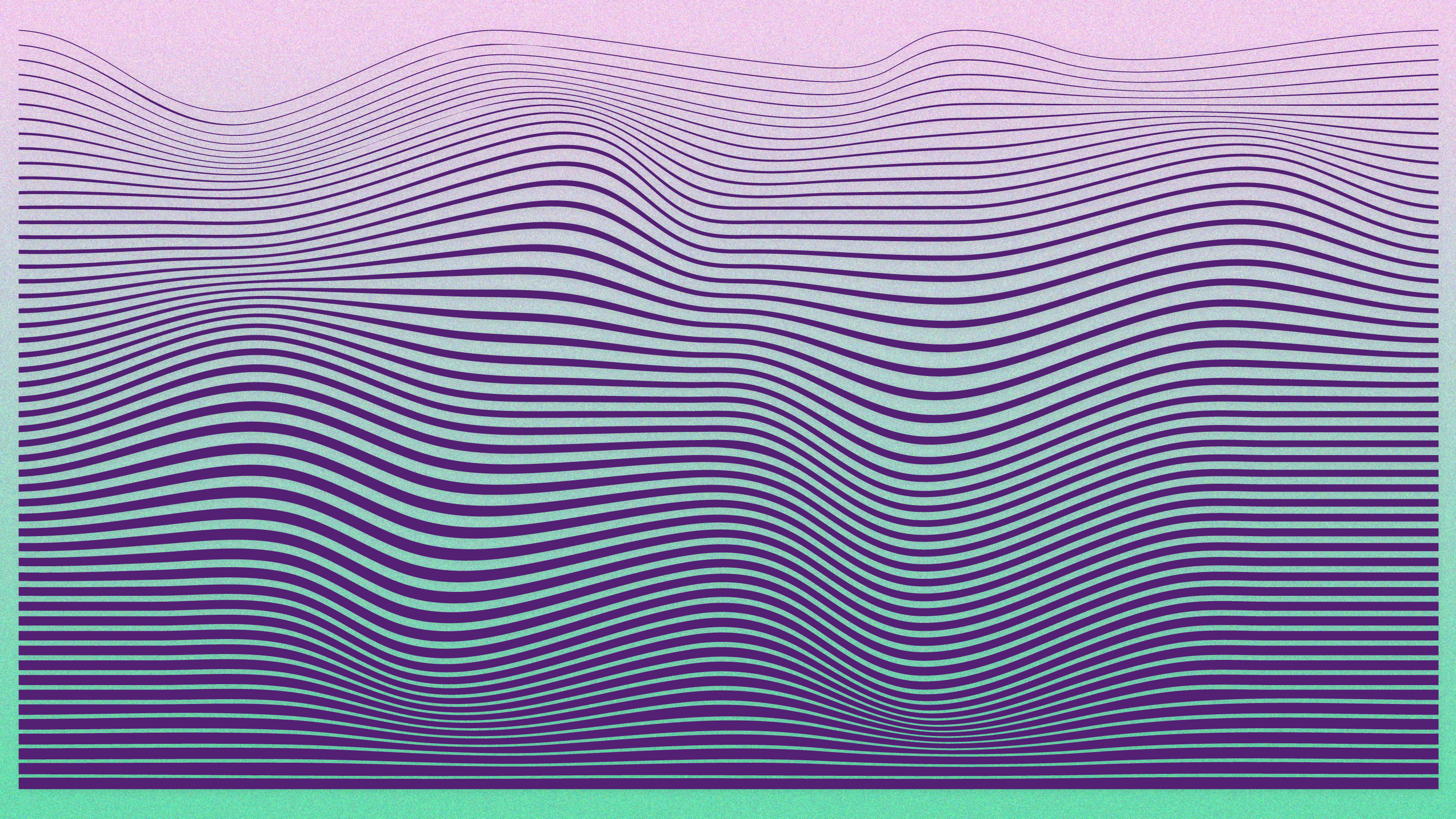

Supplement: Supplementary file 1 [file Image1.jpeg]
